# A Stakeholder Survey on Live Bird Market Closures Policy for Controlling Highly Pathogenic Avian Influenza in Vietnam

**DOI:** 10.3389/fvets.2017.00136

**Published:** 2017-08-22

**Authors:** Thi Thanh Thuy Nguyen, Lyle Fearnley, Xuan Tung Dinh, Thi Tram Anh Tran, Trong Tung Tran, Van Trong Nguyen, Damian Tago, Pawin Padungtod, Scott H. Newman, Astrid Tripodi

**Affiliations:** ^1^Emergency Center for Transboundary Animal Diseases, Food and Agriculture Organization of the United Nations, Ha Noi, Vietnam; ^2^Singapore University of Technology and Design, Singapore, Singapore; ^3^National Institute of Animal Sciences, Ha Noi, Vietnam; ^4^Institute of Economics and Finance, Ha Noi, Vietnam; ^5^Department of Livestock Production, Ministry of Agriculture and Rural Development, Ha Noi, Vietnam; ^6^Emergency Center for Transboundary Animal Diseases, Food and Agriculture Organization of the United Nations, Regional Office for Asia and the Pacific, Bangkok, Thailand; ^7^Animal Health Service, Food and Agriculture Organization of the United Nations, Rome, Italy

**Keywords:** avian influenza, live bird market, market closure, trader opinion, poultry value chain, risk mitigation

## Abstract

Extensive research in Vietnam and elsewhere has shown that live bird markets (LBMs) play a significant role in the ecology and zoonotic transmission of avian influenzas (AIs) including H5N1 and H7N9. Vietnam has a large number of LBMs reflecting the consumer preferences for live poultry. Under pressure to mitigate risks for H7N9 and other zoonotic AIs, Vietnam is considering, among other mitigation measures, temporary closures of LBMs as a policy to reduce risk of AI outbreaks. However, the efficacy of market closure is debated, particularly because little is known about how poultry traders may react, and whether trading may emerge outside formal marketplaces. Combining efforts of anthropologists, economists, sociologists, and veterinarians can be useful to elucidate the drivers behind poultry traders’ reactions and better understanding the barriers to implementing risk mitigation measures. In this paper, we present results from a stakeholder survey of LBM stakeholders in Vietnam. Our qualitative data show that trading outside formal markets is very likely to occur in the event of a temporary LBM market closure. Our data show that the poultry value chain in Vietnam remains highly flexible, with traders willing and able to trade poultry in many possible locations. Our results indicate that simplification of the poultry value chain along with strict enforcement, engagement of stakeholders, and adequate communication would be a necessary prerequisite before market closure could be an effective policy.

## Introduction and Purpose

A number of studies point to the significance of live bird markets (LBMs) in the maintenance, transmission, and spread of avian influenza (AI) viruses in poultry populations, and highlight the role of LBMs in transmission of zoonotic influenza viruses to human populations (1–7). Studies in Vietnam have shown that LBMs are at high risk for presence of AI viruses (8), and market practices were significantly associated with AI virus contamination (9–11). In China, epidemiological studies have indicated that exposure to live poultry or contaminated environments, especially markets where live birds are sold, were significant risk factors for influenza A (H7N9) infection in human (6, 12). In a number of Chinese cities, government authorities have closed live poultry markets as part of the effort to control the epidemic.^1^

Emerging subtypes or clades of highly pathogenic avian influenza (HPAI) viruses could be detected in Vietnam months or years after similar viruses were detected in China, e.g., H5N1 clade 1 and clade 2.3.2.1a and H5N6. Cross-border trade in poultry is suspected to be an important mechanism for the introduction of new zoonotic and HPAI viruses into Vietnam (14). The government of Vietnam is considering closure of LBMs as a possible emergency intervention if H7N9 or other zoonotic influenza viruses are detected in the market or in a person who has visited the market. Besides reducing direct contact between poultry and people, temporary market closure would enable cleaning and disinfection aimed at reducing virus accumulation, amplification, and spread among poultry population and transmit to humans. Market closure would include culling and disposal of all poultry on the day of closure, and the prohibition of holding or selling poultry in the market for 7 days while cleaning and disinfection would be conducted [Vietnam Ministry of Agriculture and Rural Development Action Plan (2014) on Emergency Response to Dangerous Avian Influenza Virus Strains with Potential Infection on Humans].

Although scientific consensus exists about the role of LBMs in the ecology of AI, significant debate remains about whether closure of LBMs will effectively transform this viral ecology and reduce risk of AI transmission. Both qualitative and quantitative studies of China’s 2013 LBM closures suggested that they were effective in reducing the number of human infections with H7N9 (15–16). Studies in Hong Kong, in particular, have shown that emergency closure can transform the ecology of AI and reduce risk of transmission (17, 18). However, Fournié and Pfeiffer (19) question whether market closure can be an effective long-term strategy or can be utilized in resource-poor settings. In particular, they suggest that closure may not be as effective in a future epidemic if informal marketing channels develop. Parallel informal trade routes could spread the virus to new locations, transform the structure of viral transmission networks, and worst of all, render existing targeted surveillance and risk management activities less effective (13). Fournié and Pfeiffer (19) highlight the importance of assessing the feasibility of closing markets and the likelihood of unintended adverse results, before implementing such a measure.

Although previous studies analyzed the natural ecology of poultry and AI viruses in LBMs, they left unanswered this fundamental question about the feasibility of market interventions: how will poultry traders and LBM market managers respond to market interventions, including temporary market closure? This study aims to answer this question through a qualitative, participatory survey of the perceptions and opinions of LBM stakeholders toward disease risk in LBMs and toward temporary market closure as part of government risk mitigation interventions.

Previous surveys in LBMs focused on hygiene practices and risk behaviors (20), quantifying trader scale, and analyzing market chains (9, 11, 21), but provided minimal information about the perceptions and motivations of traders or market managers. To date, a few studies have analyzed social and cultural factors impacting the ecology of AI, focusing on farmers (22, 23) or consumers (24). This study fills this remaining gap in the understanding of social and cultural factors that are relevant for the ecological dynamics of AI through an in-depth survey of the perceptions and opinions of poultry traders and market managers in LBMs, with a particular focus on their perceptions toward temporary market closures. The aim of this study is to provide policy makers with field evidence for developing adequate risk mitigation policies in response to new introductions or detection of zoonotic AI viruses.

## Methodology

### Prospective Participatory Stakeholder Research

The study employed a participatory stakeholder approach that investigates perceptions and opinions of stakeholders about problems and policies (25). Participatory research can be defined as “systematic inquiry, with the collaboration of those affected by the issue being studied, for the purposes of education and taking action or effecting change” (26). Previous research has shown that early stakeholder involvement in the response to an environment or health problem is more effective in terms of reducing negative impact and adverse reactions than *post hoc* surveys of stakeholder reactions to a policy intervention (25, 27). In this study, we adopted a prospective approach by surveying the opinions and perceptions of stakeholders in advance of policy implementation.

The study was designed by an interdisciplinary team of trained anthropologists and sociologists, including both Vietnamese and international researchers, and in consultation with experts in animal health and AI. Guiding questions were prepared and pretested in one of the LBMs in Ha Noi, which are similar in structure and trading operations with LBMs in survey areas. The piloting markets were excluded from the survey. The interviews conducted by a team of three researchers with in-depth experience in participatory survey methods. The interviewers exchanged information at the end of each interview day in the field to ensure consistency of the field interviews. Questions were addressing aspects related to market closures such as reaction of stakeholders, impact of market closure on the livelihoods, other trading options for poultry in case of market closure, reaction on compulsory culling of poultry, and willingness of stakeholders to collaborate and under which conditions traders would comply with government policy on market closure. Interviews were conducted from February to March 2014. In total, 91 face-to-face interviews were conducted with poultry wholesalers, middlemen, transporters, and retailers. The interviewees were selected randomly in the survey markets. Notes were taken by the interviewers, and data were subsequently analyzed by coding of interviews. Interviews were also conducted with market management boards in six of eight LBMs. Two markets did not have market management boards, as they were open street markets.

During interviews, stakeholders were presented with the possibility that markets would be closed by the government for a temporary period of time, either 7 or 21 days, based on the Vietnam action plan on emergency response to dangerous AI virus strains with potential infection on humans. Under this plan, the decision between 7 and 21 days closure should be based on the magnitude of the disease situation. While a closure of 7 days would apply as emergency control response in markets in a small geographic area, a 21-day closure would come into force in case of geographic spread of the disease and would include markets in a wider geographic area. These stakeholder groups were defined as follows: (1) a *market manager* appointed by local government to manage the market; (2) a *wholesaler* trades a *high* volume of poultry, primarily purchasing from farms and selling to other traders; (3) a *middleman* trades a *small to medium* volume of poultry, purchased from wholesalers and sold to other traders; (4) a *retailer* trades a *small* volume of poultry and sells directly to the end user (consumer); and (5) a *consumer* is a purchaser and end user of poultry.

### Research Setting: The LBMs

Interviews were conducted in eight LBMs in four provinces: Ha Vi and Bac Thang Long markets (Ha Noi), Re Market (Hai Phong), Dam Chieu (Hai Phong), Tuc Duyen (Thai Nguyen), Ba Hang (Thai Nguyen), Gieng Vuong (Lang Son), and That Khe (Lang Son). The markets were selected to represent diversity in scale, management, trading operations, and mode of construction, which would need to be considered by government interventions in case of HPAI outbreaks.

The term “traders” (Table 1) includes live poultry wholesalers, middlemen, and retailers. The number of live poultry traders in each market range from 10 to 190, with a mean of 62 and a median of 57. The largest market is Ha Vi market, with 190 wholesalers and middlemen trading over 30,000 birds per day.

**Table 1:** Number of traders in survey markets.

Market name	Location	Number of traders
Ha Vi	Ha Noi city	190
Bac Thang Long	Ha Noi city	60
Re market	Hai Phong city	13
Dam Chieu	Hai Phong city	10
Tuc Duyen	Thai Nguyen province	45
Ba Hang	Thai Nguyen province	55
Gieng Vuong	Lang Son province	67
That Khe	Lang Son province	59

All the surveyed markets operate 7 days per week. Three out of the eight markets are sheltered or roofed and enclosed. Traders with permanent stalls in the market pay a monthly hygiene fee of about 50,000 VND^2^ to the market management board, which hires cleaners to clean the market at the end of each day. In addition, investments have been made in two of these markets to improve the hygienic situation. For example, Ha Vi market was built during 2007–2011 with funds from the World Bank through the Vietnam Animal and Human Influenza Control and Preparedness (VAHIP) project. The VAHIP invested in a waste water treatment and drainage system. However, in all three of these markets, drainage systems remained clogged by solid wastes and therefore ineffective.

Four of the eight markets located on open streets. In these street markets, traders pay a daily market fee of about 3,000–5,000 VND^3^ per trader. Some of these street markets are nearby to official, enclosed marketplaces that do not sell live poultry. One market is neither indoors nor on a public street, but on an area of barren land. When it rains, the ground turns to mud.

## Results

In response to the possible 7-day market closure, all stakeholders pointed to the likelihood that parallel trading outside the market would emerge. At the same time, responses to parallel trading diverged according to the scale of the traders’ operations. The opinion of stakeholders on market closure for 7 days is summarized in Table 2.

**Table 2:** Opinion of stakeholders on market closure for a duration of 7 days.

Stakeholder	Opinion	Reaction	Concern
Retailer	Would follow government regulation and would not trade poultry in the market	→ Would continue to *sell poultry at home, nearby streets or make door-to-door deliveries*	Worry that regulations do not apply equally to all retailers leading to business disadvantages
Middlemen	Would follow government regulation	→ Would collect poultry from other markets or directly from farms and sell to other markets or other places such as street intersections	Worry about losing trading networks
Wholesaler	Would follow regulation	→ Would stop poultry trading	Worry about losing trading networks
Market manager	Insist they would follow the regulation		Worry about retailers continuing to trade live poultry outside markets and in public streets Stress that it would be necessary to have close coordination between various levels of the government to ensure strict enforcement and monitoring

However, in response to the possible 21-day market closure, the divergence of opinions shifted. In this case, middlemen and retailers joined wholesalers in declaring that they would halt trading of live poultry altogether. They stated that with such a long, and probably widespread closure of markets, the market demand for poultry would likely decline sharply. Many traders suggested they would temporarily shift to other jobs, such as agricultural work, or trading other products (vegetables, rice, pork, kittens, puppies, etc.). In addition, they would request for exemption or reduction of taxes and other charges (market fee and charges). Market managers remained consistent in declaring they would comply with regulations, and also noted that they would request remissions of taxes or revenue charges.

Although wholesalers had declared they would halt poultry operations during both 7- and 21-day market closures, further inquiry revealed that they disagreed with compulsory culling of poultry associated with market closures in case of market closures as specified in the Vietnam action plan. Wholesalers argued that their birds have been carefully selected and have farm origin and vaccination certificates issued by animal health authorities. Therefore, they believed the birds could not be responsible for any AI outbreaks, and therefore should not be culled. If the authorities forcibly cull poultry, wholesalers argue that they should be compensated according to the purchase (farm) price or at 50–70% of the birds’ market value. In addition, they called for assistance, such as preferential loans, following the end of the outbreak and the resumption of normal market activities. Middlemen and retailers also disagreed with the culling policy, in particular the culling of “healthy looking” birds. Both groups of stakeholders called on the government to compensate for any culled birds at market or farm price. Retailers also suggested that they might try to bring birds home to avoid being culled. The market managers worried that traders would protest against any culling of poultry. They suggested that any decision to cull poultry should combine strict enforcement with good communication and explanation. They noted that the government does have a mechanism for assistance and compensation in the case of poultry culls and suggested it should be used to enhance compliance of traders.

If a zoonotic influenza virus is detected in a market, the veterinary authorities will also need to go beyond local market interventions and rapidly identify the source of the infection to focus control measures at the origin. When reporting about the willingness and ability to locate the farms of origin, responses varied according to the scale and structure of trader operations. Wholesalers claimed that tracing their poultry back to the farm of origin would be easy since their poultry typically have origin and vaccination certificates issued by animal health authorities. Wholesalers would be able and willing to provide the addresses of the farms of origin.

Middlemen claimed they purchased birds both directly from farms and from other markets or street vendors. Birds purchased directly from farms would be easy to trace, but it would be difficult or impossible to trace birds purchased at markets or on roads. Finally, retailers suggested that it would be difficult or impossible to trace the origin farm of their birds, because they purchase birds from different sources.

## Discussion: Value Chain Flexibility and the Ecology of AI

Research in Vietnam indicates that markets connected through trade networks can contribute to large-scale epidemics, while providing opportunities for effective control as well. Targeting network hubs for surveillance, hygiene and biosecurity interventions at LBMs could reduce the transmission of virus through the network (11, 28) for China (4). The results of our study reveal that despite their position as hubs in trade networks, temporary “emergency” market closures of 7 days in case of new detection of AI viruses are unlikely to reduce the spread of AI viruses. Poultry traders, in particular middlemen and retailers, maintain a highly flexible practice of market transactions along the poultry value chain. The physical location of the LBM is only one among many possible transaction sites. Our results showed that temporary market closure for 7 days is likely to lead to establishment of parallel, informal, and uncontrolled live poultry trade, which could lead to virus introduction into non-affected areas (29). Our study concludes that given the structure of Vietnam’s poultry value chain, which remains highly flexible with numerous middlemen between producer and consumer, closure of LBMs, unless implemented on a longer term and in a larger geographical area or nationwide, will be an ineffective strategy for reducing the risk of AI. However, decisions on longer term closures, would need to take into consideration the economic effects on the poultry sector (30). The importance of timely and appropriate compensation following simple procedures for culled birds appeared consistently among the responses of all the stakeholders. While current Vietnam government regulations do foresee financial compensation for compulsory culling of poultry at farms, there is at present no provision for compensation for poultry culled at markets. If stakeholders do not perceive culling as a justified measure, they will be more prone to disobey regulations and trade their poultry through unofficial channels. Proper communication of compensation schemes has shown to be crucial to improve compliance and avoid unintended effects (31, 32).

The results of our study demonstrated that poultry value chains in northern Vietnam contain a high degree of flexibility. In an agricultural value chain, “actors are connected along a chain producing, transforming, and bringing goods and services to end-consumers through a sequenced set of activities” (33). Value chain analysis has tended to provide formalistic accounts of market relationships, focusing on the vertical links that bring a product “from farm to fork.” We propose the concept of the “flexibility” of the value chain to describe the capacity of a value chain to shift spatially, or to forge new transaction links, in the event that a particular site or relationship of exchange is eliminated (e.g., through market closure). Our study exposed the high degree of flexibility of the value chain in Vietnam. The flexibility of market transactions far exceeds the physical space of the marketplace, i.e., the LBM. If the LBM is closed for 7 days, trading would continue in other forms and locations. At the same time, this flexibility itself was not shared equally by all stakeholders. Wholesalers, due to the large scale of their operations, were more closely bound to the institutional setting and physical site of the formal LBM marketplace. Furthermore, they reported that they purchase poultry with official certification of farm of origin, indicating a relatively stable and traceable part of the value chain. Small retailers, by contrast, purchase birds from middlemen or wholesalers each day and sell them again, often on the side of small streets or by delivering them to small restaurants. Middlemen buy and sell poultry with the highest degree of flexibility: they report that they could easily shift operations to other markets in the event of market closure for 7 days. The restructuring and simplification of the poultry value chain as suggested by several poultry value chain studies conducted in Vietnam (FAO unpublished data), by reducing the number of middlemen and small-scale traders, could decrease the overall flexibility of trading and therefore improve the effectiveness of market closure. The flexibility of the poultry value chain explains why market closure may not be an effective strategy for reducing the spread of AI or AI incursion risk. The closure of the marketplace is intended to eliminate a key node in the network of AI transmission. But LBM traders do not necessarily confine their trading to the LBM. As traders exploit the flexibility of the value chain and shift transactions to parallel trading sites, live poultry trading networks may expand and fragment, increasing rather than reducing AI transmission and risk.

Finally, our results also revealed important limits to the flexibility of the poultry value chain. A minor limit exists in the length of market closure. During a 7-day closure, all stakeholders described how they would adapt by shifting market operations to other locations, but during closure of minimum 21 days, all stakeholders reported that they would halt trading operations. Rather than trading live poultry in alternate locations along the value chain, they reported that they would shift to other forms of economic activity: trading non-poultry products or even returning to farm work. However, prolonged market closures may result in high economic losses and impact livelihoods in the poultry production sector (34).

## Conclusion

The present study shows that analyzing perceptions of stakeholders regarding risk mitigation interventions, such as the temporary closures of markets, are crucial for the design of effective policies and to avoid adverse results.

To date, the implementation of market closures has been based on viral surveillance data relying on virus detection alone. Neglecting the fact that LBMs are human, social and cultural institutions may render disease control policy ineffective. In fact, the role of LBMs in the chain of influenza transmission is conditioned by the practices and perceptions of LBM stakeholders. As a result, although market places may be closed, marketing practices and networks may continue to operate in a shifted form and facilitate AI virus spread. The position of LBMs in the poultry value chain in the North of Vietnam exemplify how the natural ecology of AI is shaped as a consequence of human perceptions and reflexive practices (35). In such cases, understanding the ecology of the virus and how to manage its risks relies on understanding the human stakeholders that construct, and can unexpectedly reconstruct, the links in the chains of viral transmission.

Stakeholder participation should be an integral part of the development of science-based policy interventions, not only for reasons of equity and ownership but also more importantly to provide accurate knowledge about natural ecology itself (25) and to ensure planning and implementation of more effective risk mitigation measures.

## Ethics Statement

The study was considered low risk as the main risks to study respondents were believed to be abreact of confidentiality and privacy. To mitigate these risks, the following safeguards were put in place. Verbal informed consent was received from study participants during the recruitment process. Another verbal informed consent was received from participants before each interview or group discussion after clear explanation about the objectives and content of the study. Each study participant was assigned a code to maintain confidentiality during data collection and analysis. No personal identifiers, including names, were collected at any time throughout the study.

## Author Contributions

All the authors: substantial contributions to the design of the study, collecting data in the field, and analysis and interpretation of the data; drafting the report; approval of the version to be published; and agreement to be accountable for all aspects of the manuscript.

## Conflict of Interest Statement

The authors declare that the research was conducted in the absence of any commercial or financial relationships that could be construed as a potential conflict of interest.

## Footnotes

^1^The strategy of live bird market closure is based in part on the earlier experiences of Hong Kong. The 1997 outbreak of H5N1 in Hong Kong continues to provide a standing example of the potential for reduction or even eradication of avian influenza viruses through closure of markets coupled with poultry culls. However, as the Hong Kong case also demonstrates, the ability to eradicate AI from one city or region does not ensure protection against the later reintroduction of the virus (13).

^2^Equivalent to 2.2 USD.

^3^Equivalent to 0.12–0.22 USD.
